# Pregnancy incidence and intention after HIV diagnosis among women living with HIV in Canada

**DOI:** 10.1371/journal.pone.0180524

**Published:** 2017-07-20

**Authors:** Kate Salters, Mona Loutfy, Alexandra de Pokomandy, Deborah Money, Neora Pick, Lu Wang, Shahab Jabbari, Allison Carter, Kath Webster, Tracey Conway, Daniele Dubuc, Nadia O’Brien, Karene Proulx-Boucher, Angela Kaida, on behalf of the CHIWOS Research Team

**Affiliations:** 1 Faculty of Health Sciences, Simon Fraser University, Burnaby, Canada; 2 British Columbia Centre for Excellence in HIV/AIDS, Vancouver, Canada; 3 Women's College Research Institute, Women's College Hospital, University of Toronto, Toronto, Ontario, Canada; 4 Chronic Viral Illness Service, McGill University Health Centre, Montreal, Quebec, Canada; 5 Department of Family Medicine, McGill University, Montreal, Quebec, Canada; 6 Oak Tree Clinic, BC Women’s Hospital and Health Centre, Vancouver, Canada; 7 Faculty of Medicine, University of British Columbia, Vancouver, Canada; University of Washington Department of Global Health, UNITED STATES

## Abstract

**Background:**

Pregnancy incidence rates among women living with HIV (WLWH) have increased over time due to longer life expectancy, improved health status, and improved access to and HIV prevention benefits of combination antiretroviral therapy (cART). However, it is unclear whether intended or unintended pregnancies are contributing to observed increases.

**Methods:**

We analyzed retrospective data from the Canadian HIV Women’s Sexual and Reproductive Health Cohort Study (CHIWOS). Kaplan-Meier methods and GEE Poisson models were used to measure cumulative incidence and incidence rate of pregnancy after HIV diagnosis overall, and by pregnancy intention. We used multivariable logistic regression models to examine independent correlates of unintended pregnancy among the most recent/current pregnancy.

**Results:**

Of 1,165 WLWH included in this analysis, 278 (23.9%) women reported 492 pregnancies after HIV diagnosis, 60.8% of which were unintended. Unintended pregnancy incidence (24.6 per 1,000 Women-Years (WYs); 95% CI: 21.0, 28.7) was higher than intended pregnancy incidence (16.6 per 1,000 WYs; 95% CI: 13.8, 20.1) (Rate Ratio: 1.5, 95% CI: 1.2–1.8). Pregnancy incidence among WLWH who initiated cART before or during pregnancy (29.1 per 1000 WYs with 95% CI: 25.1, 33.8) was higher than among WLWH not on cART during pregnancy (11.9 per 1000 WYs; 95% CI: 9.5, 14.9) (Rate Ratio: 2.4, 95% CI: 2.0–3.0). Women with current or recent unintended pregnancy (vs. intended pregnancy) had higher adjusted odds of being single (AOR: 1.94; 95% CI: 1.10, 3.42), younger at time of conception (AOR: 0.95 per year increase, 95% CI: 0.90, 0.99), and being born in Canada (AOR: 2.76, 95% CI: 1.55, 4.92).

**Conclusion:**

Nearly one-quarter of women reported pregnancy after HIV diagnosis, with 61% of all pregnancies reported as unintended. Integrated HIV and reproductive health care programming is required to better support WLWH to optimize pregnancy planning and outcomes and to prevent unintended pregnancy.

## Introduction

Health and survival outcomes for people living with HIV have improved dramatically since the advent of combination antiretroviral therapy (cART) [1–3]. HIV treatment with sustained viral suppression is also enabling safer reproductive options for women living with HIV (WLWH), including better maternal health, improved fertility [4], negligible risk of HIV transmission to partners during condomless sex [5–7], and dramatic reductions in perinatal HIV transmission risk [8–10]. These improvements have transformed the reproductive health landscape for people living with or affected by HIV [11–17], with studies demonstrating that WLWH are more likely to become pregnant and have children in the modern cART era than in earlier years of the HIV epidemic [18, 19].

Among WLWH in Canada, cART use is associated with increased fertility intention and an increase in the proportion of WLWH becoming pregnant over time [12]; a finding that has been corroborated in other settings [15, 18–23]. However, it is unknown whether such increases in pregnancy incidence are driven by intended or unintended pregnancies [13, 15, 24–26], raising questions about how women’s reproductive health and agency are prioritized and supported after an HIV diagnosis.

Supporting WLWH to plan for desired pregnancy and prevent unintended pregnancy is a critical component of comprehensive women-centred HIV care [27] and central to women’s sexual and reproductive health and rights and HIV prevention efforts [8, 28–32]. Unintended pregnancies have numerous potential health implications including, higher risks of maternal and infant morbidity and mortality, delayed engagement in antenatal care, poorer birth outcomes, and poorer retention in postpartum follow-up [33]. Such risks are exacerbated for WLWH, for whom early engagement in pre-conception, antenatal, and postpartum care is key to maximizing maternal health and minimizing risks of perinatal transmission [34]. A pregnancy after an HIV diagnosis can be a stressful time, with women expressing significant worry and fear about HIV-related concerns (i.e., their own health and the health of their infant) as well as non HIV-related concerns (i.e., financial and relationship stress), coupled with experiences of internalized and experienced HIV-related stigma around becoming pregnant [35, 36]. Determining the contribution of unintended pregnancy to the overall incidence of pregnancy among WLWH is a key step towards the development of comprehensive reproductive rights- and evidence-based programming across the continuum of reproductive care including pre-conception care when pregnancy is intended, contraceptive access when it’s not, and the availability of safe termination services.

The primary objective of this study was to measure cumulative incidence and incidence rate of pregnancy after HIV diagnosis among a cohort of WLWH in Canada overall and by pregnancy intention, cART use status at time of pregnancy, and cART era (before or after the year 2000). Our secondary objective was to determine independent socio-demographic and clinical correlates of unintended pregnancy among the most recent or current pregnancy. This information is key to the provision of targeted and tailored reproductive health services for WLWH, to mitigate health risks, support reproductive rights, and focus prevention efforts more effectively.

## Methods

### Study setting

As of 2011, there were approximately 16,600 WLWH in Canada, an increase of nearly 13% from three years prior [37]. The majority of WLWH reside in the three Canadian provinces of British Columbia, Ontario and Quebec. HIV prevalence and incidence are inequitably distributed among women in Canada along several social axes, including poverty, Indigenous ancestry, injection drug use and/or sex work histories, refugee and newcomer status, African, Caribbean, or Black Canadian (ACB) ethnicity, and sexual and gender identities, with several points of intersection between and within these groups [37].

### Study design and guiding frameworks

This analysis used baseline questionnaire data from the Canadian HIV Women’s Sexual and Reproductive Health Cohort Study (CHIWOS), a multi-site, community-based research study conducted by, with, and for WLWH in collaboration with researchers, healthcare providers, policy-makers, and other stakeholders. As described in detail elsewhere [27, 38], the primary objectives of CHIWOS are to assess the prevalence, barriers, and facilitators to use of women-centred HIV care, and the impact of such patterns of use on health outcomes. CHIWOS is grounded in community-based research principles [39], and guided by Social Determinants of Health [40, 41] and Critical Feminism [42] frameworks, with the aim of producing community-driven research and action on health priorities for WLWH.

### Study population, recruitment, and procedures

Eligible participants included WLWH (self-identified) aged ≥16 years, including trans and gender diverse women, residing in British Columbia, Ontario, and Quebec. Between August 27, 2013 and May 1, 2015, women were recruited to participate in CHIWOS through peer word-of-mouth, HIV clinics, AIDS Service Organizations, non-HIV community-based organizations, the networks of our national Steering Committee and three provincial Community Advisory Boards, and online methods such as listservs for WLHIV and our study’s website (www.chiwos.ca), Facebook (www.facebook.com/CHIWOS), and Twitter (www.twitter.com/CHIWOSresearch) pages [43]. At enrolment, participants completed structured, comprehensive questionnaires in English or French using online FluidSurveys^™^ software [44], administered by Peer Research Associates, who are WLWH who completed training in community-based research methods [45]. Study visits took 1.5 to 2.5 hours and were completed either in-person at collaborating HIV clinics, AIDS Service Organizations, or women’s homes, or via phone/Skype for those living in rural and remote areas. Follow-up interviews at 18-month intervals are in-progress.

Participants provided voluntary, informed consent either by signing a consent form or, if questionnaires were completed remotely, via verbal consent with a witness. Ethical approval for this study was obtained from the Research Ethics Boards of Simon Fraser University, University of British Columbia/Providence Health, Women’s College Hospital, and McGill University Health Centre.

### Analytic sample: Inclusion and exclusion criteria

A total of 1,424 WLHIV enrolled in the CHIWOS study and completed the baseline visit. Baseline questionnaire data were used for this analysis including reports of lifetime history of pregnancy (up to eight pregnancy events in a lifetime). For this analysis, we excluded participants who did not identify their biological sex as female (n = 57), women aged 45 or older at HIV diagnosis (n = 139), women who reported completing menopause before HIV diagnosis (n = 16), and women with unknown HIV diagnosis year (n = 47), yielding an analytic sample of 1,165 women. We further restricted pregnancy incidence analyses to women who were diagnosed with HIV prior to date of pregnancy (estimated at conception). For pregnancy incidence analyses and time-to-event calculations, we also excluded 19 participants missing pregnancy dates and 10 who reported a pregnancy end date that was the same as the HIV diagnosis date. These exclusions yielded an analytic sample of 1,136 women. Finally, for the analysis of correlates of unintended pregnancy, we restricted the analysis to the most recent or current pregnancy reported after HIV diagnosis.

### Measures

#### Primary outcome: Pregnancy incidence after HIV diagnosis

We assessed total number of lifetime pregnancies and detailed retrospective event-level data for all reported pregnancies, up to a maximum of eight lifetime pregnancies. The primary outcome was all self-reported pregnancy events occurring after HIV diagnosis until censored (CHIWOS interview date), including current pregnancies. For each pregnancy event reported after HIV diagnosis, data were captured on pregnancy outcome (single live birth, multiple live births, miscarriage, still birth, pregnancy termination, or ectopic pregnancy), pregnancy intention (unintended vs. intended vs. don’t know), HIV status of the other biological parent (HIV positive vs. HIV negative vs. unknown), on cART before pregnancy occurred (yes vs. no), date of pregnancy outcome (month and year), duration of pregnancy, and, if applicable, infant HIV status (HIV-positive, HIV-negative, testing underway, or unknown). We estimated date of conception by subtracting the duration of pregnancy from the date of pregnancy outcome.

To assess the pregnancy incidence rate, we applied the following rules to calculate woman-years (WY) ‘at risk’ for pregnancy: (1) Time of follow-up began at reported HIV diagnosis date or at 16 years of age, whichever date was later to avoid biasing time at risk among perinatally infected women and (2) Women were censored at age 45, date of surgically induced menopause (hysterectomy), or date of CHIWOS interview, whichever occurred first.

For analyses focused on the most recent or current pregnancy, we assessed additional information regarding the pregnancy experience, including ‘feeling when found out about the current or most recent pregnancy’ (very happy/happy vs. not sure/unhappy/very unhappy vs. unknown), ‘happiness during current or most recent pregnancy’ (happiest time/happy time vs. moderately hard/hard/worst times vs. unknown) [46]. Considering these additional data, we only captured most recent or current pregnancy in the bivariate and multivariable analyses.

#### Explanatory variables

We examined associations of pregnancy incidence after HIV diagnosis and unintended pregnancy with baseline variables, identified *a priori* from the established literature [11, 12, 20, 23, 25, 26]. Socio-demographic variables included: age at interview [median and interquartile range (IQR)], province of interview (British Columbia vs. Ontario vs. Quebec), ethnicity (Indigenous vs. African/Caribbean/Black vs. White vs. other ethnicities), whether the participant was born in Canada (yes vs. no), legal relationship status at interview (married/common-law vs. single), sexual orientation [heterosexual vs. lesbian, gay, bisexual, two-spirit or queer (LGBTQ)], education (<high school vs. ≥high school), annual personal income at interview (<$20,000 vs. ≥$20,000 per year), history of injection drug use (yes vs. no) and history of incarceration (yes vs. no).

Health-related variables included Hepatitis C (HCV) co-infection (yes vs. no) and lifetime diagnosis of any mental health condition (yes vs. no). HIV clinical variables included current cART use (current use vs. previous use vs. never) and years living with HIV at interview. We defined cART initiation era as ‘before the year 2000’ or ‘2000 or later’ as this is a general mark of when therapy guidelines shifted to include evidence on the benefits of cART [47, 48].

### Statistical analysis

For the primary objective, we assessed baseline characteristics of the sample of 1,165 WLWH including pregnancy event information, demographics, and HIV diagnosis information. We used Kaplan-Meier (K-M) methods to estimate cumulative incidence of time to first pregnancy after HIV diagnosis or age 16 (whichever came later) to CHIWOS interview date. Overall cumulative incidence of first pregnancy after HIV diagnosis was calculated as well as by reported pregnancy intention (intended vs. unintended), by cART status (on cART vs. not on cART prior to the start of pregnancy), and by cART era (prior to the year 2000 vs. the year 2000 until 2015). Log-rank test assessed statistical differences in the K-M curves.

Generalized estimating equation (GEE) Poisson models were used to calculate pregnancy incidence rates expressed as incidence rate per 1,000 women-years (WY) with 95% confidence intervals overall, by pregnancy intention, and cART status.

For the secondary objective, baseline characteristics of study participants were compared for women with reported recent or current pregnancy by pregnancy intention, using Pearson χ^2^ test or Fisher’s exact test for categorical variables and Wilcoxon’s Rank Sum test for continuous variables. Multivariable logistic regression was used to identify independent correlates of unintended pregnancy of the most recent or current pregnancy. Model selections were conducted using a backward stepwise elimination technique based on two criteria (Akaike Information Criterion (AIC) and Type III p-values).

## Results

### Baseline characteristics of study population

Among 1,165 WLWH included in the analysis, 30.1% identified as African/Caribbean/Black, 22.2% identified as Indigenous, 40.9% identified White and 6.8% as other ethnicities; 24.4% were from British Columbia, 51.5% from Ontario, and 24.1% from Quebec. Our sample included 12.2% LGBTQ women, 30.7% with a history of injection drug use, and 35.7% born outside of Canada. Median age at interview was 41 (IQR: 35–48) and median years since HIV diagnosis was 12 (IQR: 6–17).

### Pregnancy incidence and outcomes

Among 1,165 WLWH, 278 (23.9%) women reported a total of 492 pregnancies after being diagnosed with HIV, with 299 (60.8%) of those pregnancies reported as unintended. Of 492 pregnancies, 92 (18.7%) resulted in miscarriage or stillbirth, 101 (20.5%) were terminated, and 282 (57.3%) resulted in a single or multiple live birth. Seventeen women (3.5%) were pregnant at time of interview. Among the 282 live births, 94.7% of infants tested HIV-negative (Table 1). Among the sample of 1,165 WLWH, the median number of pregnancies from time of HIV diagnosis until interview, including miscarriage, terminations and current pregnancies, was 2 (IQR: 1–4) and the median number of children was 1 (IQR: 0–3). Of the pregnancies occurring after HIV diagnosis, the median age at conception was 30 years [IQR: 25–34] and in 328 (66.7%) of pregnancies, women had initiated cART prior to the start of pregnancy.

**Table 1:** Pregnancy events and outcomes after HIV diagnosis among 278 women living with HIV enrolled in the Canadian HIV women sexual and reproductive health cohort study (CHIWOS), n = 492.

Variable	Response	n	%
**Age at conception (n = 441)**		(median) 30	(IQR) 25–34
**Currently pregnant**	Yes	17	3.5%
**Pregnancy intention**			
	Intended pregnancy	189	38.4%
	Unintended pregnancy	299	60.8%
	Other*	<5	<1%
**Pregnancy outcome**			
	Single or multiple live birth	282	57.3%
	Miscarriage, Stillbirth, or Ectopic pregnancy	92	18.7%
	Pregnancy termination	101	20.5%
**On ART prior to pregnancy**			
	Yes	328	66.7%
	No	154	31.3%
	Other*	10	2.0%
**HIV test result of baby (live births, n = 282)**			
	HIV positive	<5	<1%
	HIV negative	267	94.7%
	Testing underway	<5	<1%
	Unknown	8	2.8%

*Other = unknown or prefer not to answer;

ART, antiretroviral therapy; IQR, interquartile range

**Fig 1:**
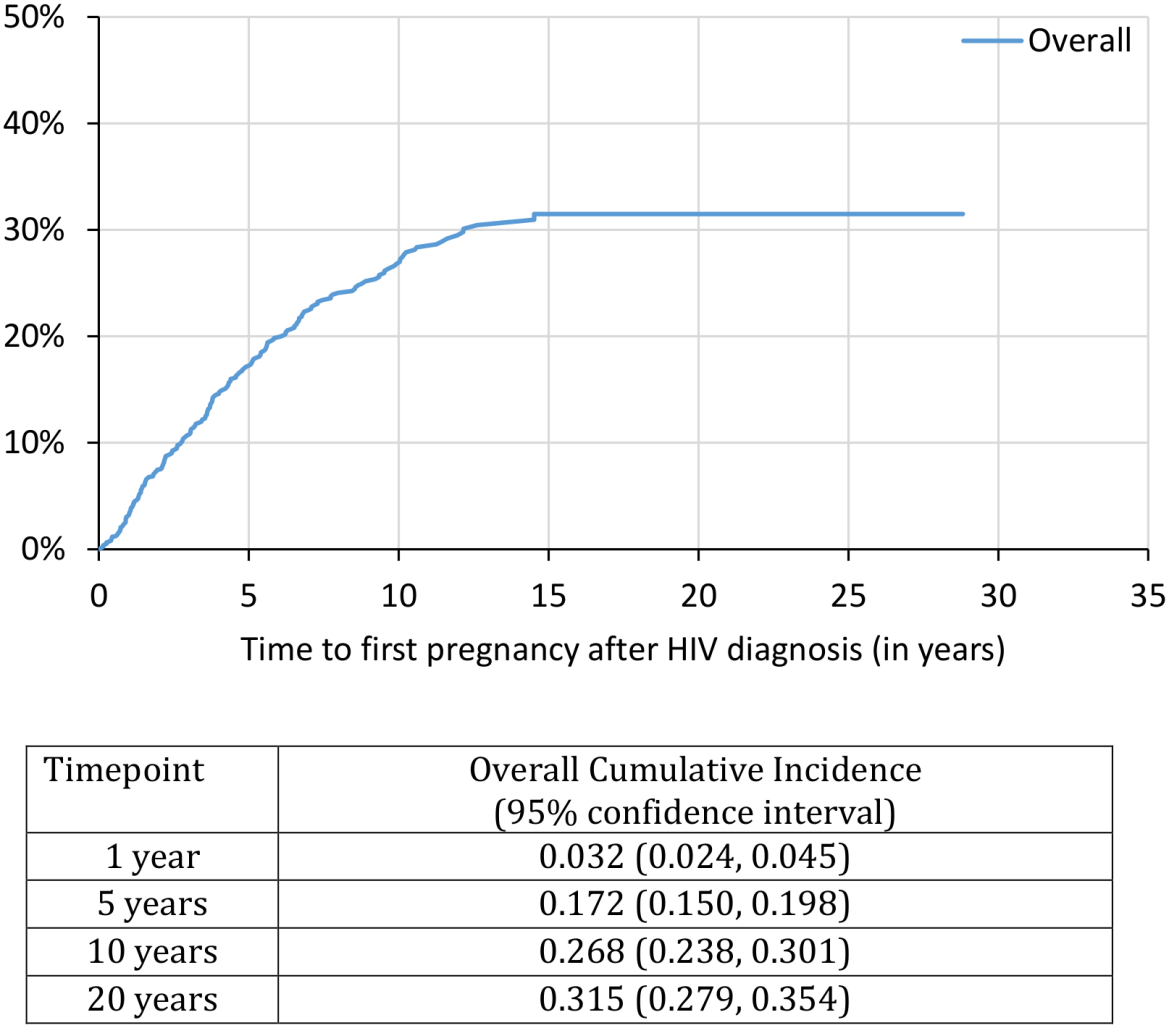
Time to first pregnancy after HIV diagnosis. This figure demonstrates the estimated time to first pregnancy event (inclusive of live births, terminations and miscarriages/stillbirths) for all women living with HIV in our sample that provided HIV diagnosis date and estimated date of pregnancy (n = 1,136). This figure demonstrates the cumulative incidence estimates and 95% confidence intervals at 1-, 5-, 10-, and 20-years for any pregnancy event.

**Fig 2:**
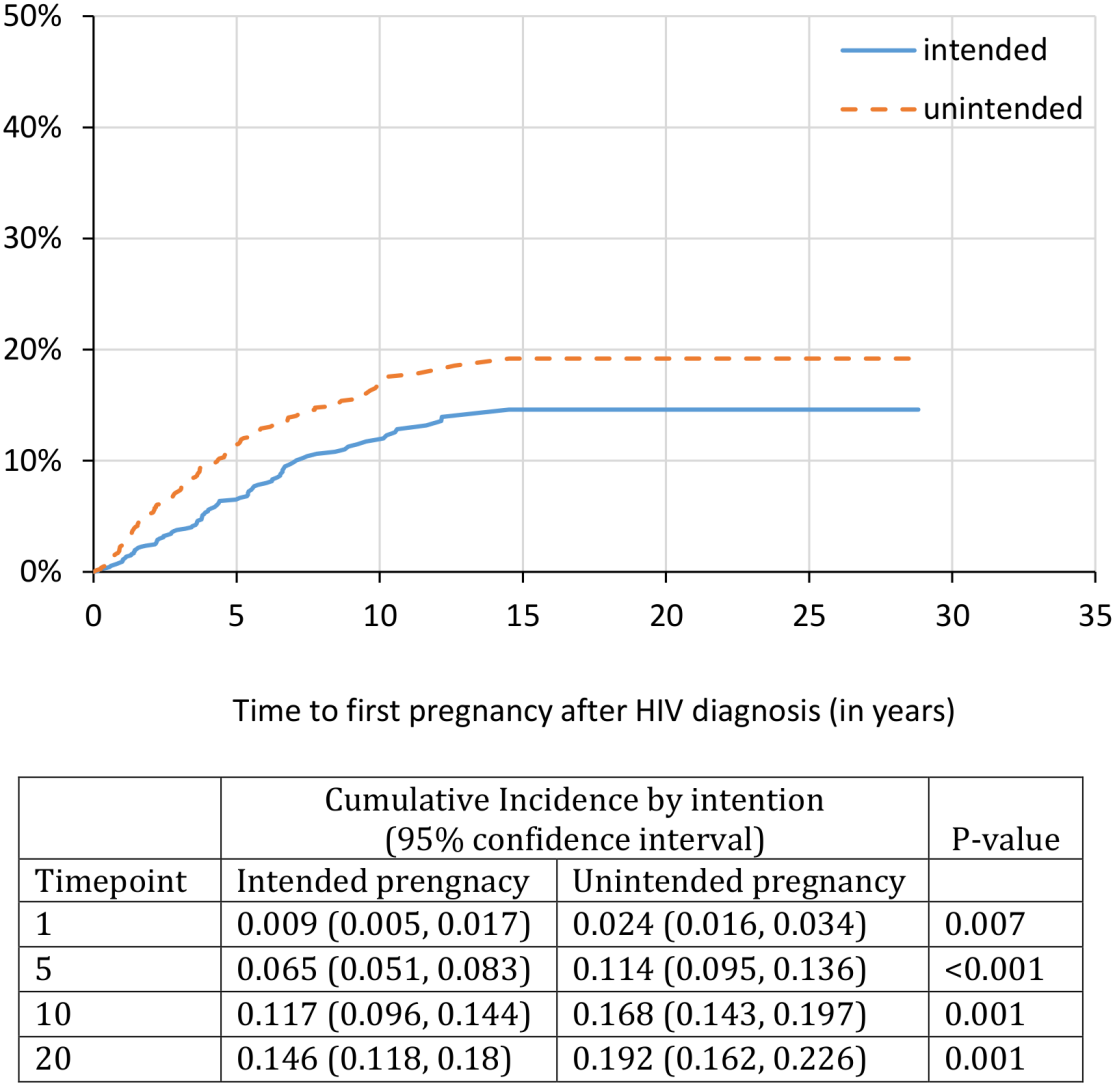
Time to first pregnancy after HIV diagnosis by intention status. This figure demonstrates the estimated time to first pregnancy event (inclusive of live births, terminations and miscarriages/stillbirths) for all women living with HIV in our sample that provided HIV diagnosis date and estimated date of pregnancy (n = 1,136). These pregnancies are stratified by self-reported intention as either intended or unintended pregnancy. This figure demonstrates the cumulative incidence estimates and 95% confidence intervals at 1-, 5-, 10-, and 20-years for unintended and intended pregnancies.

**Fig 3:**
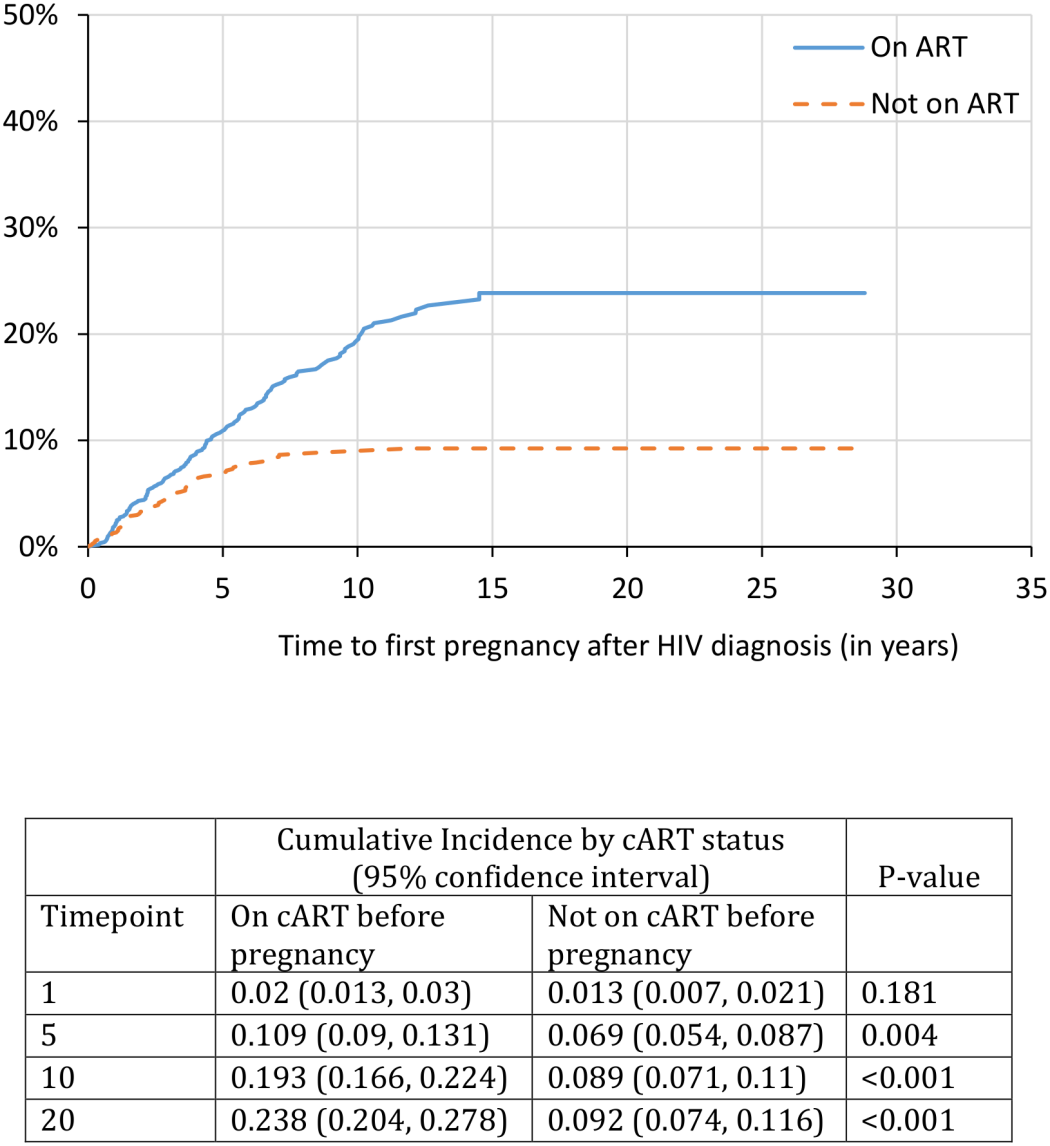
Time to first pregnancy after HIV diagnosis by cART status prior to pregnancy. This figure demonstrates the estimated time to first pregnancy event (inclusive of live births, terminations and miscarriages/stillbirths) for all women living with HIV in our sample that provided HIV diagnosis date and estimated date of pregnancy (n = 1,136). These pregnancies are stratified by self-reported uptake of cART prior to the start of pregnancy. This figure demonstrates the cumulative incidence estimates and 95% confidence intervals at 1-, 5-, 10-, and 20-years for pregnancies cART exposed and those pregnancies not exposed to cART.

**Fig 4:**
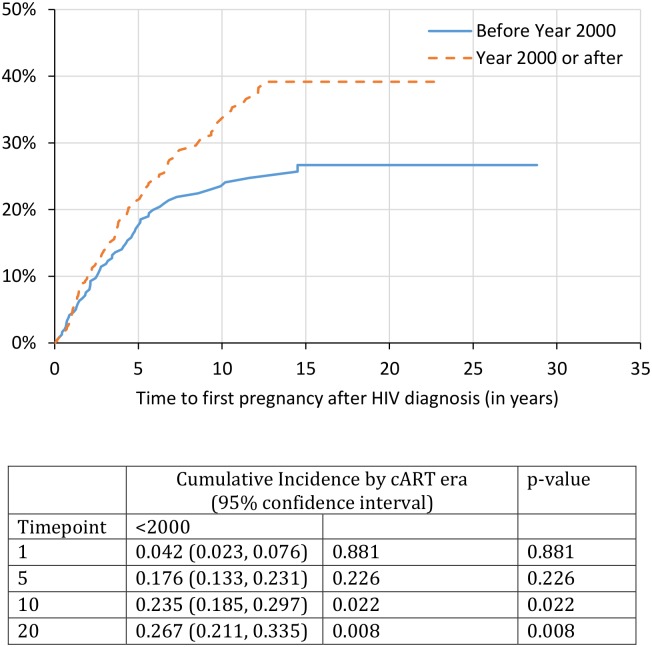
Time to first pregnancy after HIV diagnosis by era of cART initiation. This figure demonstrates the estimated time to first pregnancy event (inclusive of live births, terminations and miscarriages/stillbirths) for all women living with HIV in our sample that provided HIV diagnosis date and estimated date of pregnancy (n = 1,136). These pregnancies are stratified by self-reported date of cART initiation (prior to the year 2000 vs the year 2000 or later). This figure demonstrates the cumulative incidence estimates and 95% confidence intervals at 1-, 5-, 10-, and 20-years for all pregnancies by cART era.

Using a further restricted sample of 1,136 with HIV diagnosis and pregnancy dates provided, we identified that by 1, 5, 10, and 20 years post HIV diagnosis, the cumulative probability of first pregnancy after HIV diagnosis was 3%, 17%, 27% and 32%, respectively (Fig 1). Unintended pregnancy (vs. intended) was cumulatively higher over time; with K-M curves diverging at 1-year post HIV diagnosis (2.4% vs. 0.9%, p = 0.007), at 5 years post HIV diagnosis (11.4% vs. 6.5%, p<0.001), and at 10 years post HIV diagnosis (16.8% vs 11.7%, p = 0.001) (Fig 2). At the 20-year mark after HIV diagnosis, the cumulative incidence of first pregnancy for those with unintended pregnancy was 19.2% (95% CI: 16.2%-22.6%) compared to pregnancy incidence among those with intended pregnancy of 14.6% (95% CI: 11.8%-18.0%). Women on cART prior to pregnancy had higher probability of pregnancy over time; with K-M curves diverging at 5 years post HIV diagnosis (10.9% vs. 6.9%, p = 0.004) and at 10 years post HIV diagnosis (19.3% vs. 8.9%, p<0.001) (Fig 3). At the 20-year mark post HIV diagnosis, the cumulative incidence of first pregnancy for those on cART was 23.8% (95% CI: 20.4%-27.8%) compared to pregnancy incidence among those not on cART at time of pregnancy with prevalence of 9.2% (95% CI: 7.4%-11.6%). Lastly, while there was no difference at the 1- or 5-year mark, women who initiated cART before the year 2000 had a significantly lower probability of pregnancy at the 10-year and 20-year mark. At the 20-year mark post HIV diagnosis, the cumulative incidence of first pregnancy for those who initiated cART before the year 2000 was 26.7% (95% CI: 21.1%-33.5%) compared to those initiated therapy in the year 2000 or later (39.2% [95%CI: 33.8%-45.1%]) (Fig 4).

After excluding pregnancies with unknown delivery/termination date, 256/1,146 women reported 423 pregnancies after HIV diagnosis (incidence = 41.6 per 1,000 WYs; 95% CI: 36.6–47.2) over 10,172.5 WYs of follow-up (Table 2). Incidence of unintended pregnancy (24.6 per 1,000 WYs; 95% CI: 21.0–28.7) was significantly higher than the incidence of intended pregnancy (16.6 per 1,000 WYs; 95% CI: 13.8–20.1) after HIV diagnosis. By cART use, pregnancy incidence among WLWH on cART before or during pregnancy (29.1 per 1000 WYs; 95% CI: 25.1–33.8) was significantly higher than the pregnancy incidence among WLWH not on cART during pregnancy (11.9 per 1000 WYs; 95% CI: 9.5–14.9).

**Table 2:** GEE Poisson model to calculate pregnancy incidence rates, expressed asnumber of pregnancies per 1000 woman-years (WY) (95% confidence interval).

	Pregnancy type	Number of observations	Incidence rate per 1000 WY (95% Confidence Interval)	Rate Ratio (95% Confidence interval)
**Overall**	-	423	41.6 (36.6–47.2)	-
**Intention**	Intended	169	16.6 (13.8–20.1)	1.5 (1.2–1.8)*
	Unintended	250	24.6 (21.0–28.7)	
**cART status**	On cART before pregnancy	296	29.1 (25.1–33.8)	2.4 (2.0–3.0)*
	Not on cART before pregnancy	121	11.9 (9.5–14.9)	

*Statistically significant at p<0.001

### Correlates of most recent/current pregnancy after HIV diagnosis

Of the 265 most recent or current pregnancies, 110 (41.5%) were intended and 155 (58.5%) were unintended (Table 3), with only 62 (23.4%) pregnancies occurring prior to the year 2000 (pre-cART era). Women who reported their recent/current pregnancy as unintended compared to intended, were significantly (p<0.05) more likely to identify as Indigenous (vs White) (20.0% vs. 11.8%), born in Canada (63.9% vs. 39.1%), be currently single (60.0% vs. 45.5%), co-infected with HCV (38.7% vs. 21.8%), have a history of incarceration (45.8% vs. 26.4%), have a history of injection drug use (38.7% vs. 23.6%), initiated ART before the year 2000 (29.0% vs. 15.5%), and to report having been previously diagnosed with a mental health disorder (inclusive of depression, anxiety, post-traumatic stress disorder and addiction) (49.0% vs. 32.7%).

**Table 3:** Bivariate analysis for intended vs. unintended current or most recent pregnancy, among women living with HIV (n = 265).

Variable	Response	Intended pregnancy, n = 110 (n, %)	Unintended pregnancy n = 155 (n, %)	p-value
Province interview conducted				
	British Columbia	24 (21.8%)	46 (29.7%)	0.065
	Ontario	44 (40.0%)	70 (45.2%)	
	Quebec	42 (38.2%)	39 (25.2%)	
Ethnicity				
	Indigenous	13 (11.8%)	31 (20.0%)	0.004
	African/Caribbean/Black	57 (51.8%)	48 (31.0%)	
	White	32 (29.1%)	67 (43.2%)	
	Other ethnicities	8 (7.3%)	9 (5.8%)	
Born in Canada				
	Yes	43 (39.1%)	99 (63.9%)	<0.001
	No	67 (60.9%)	56 (36.1%)	
Current legal relationship status				
	Married/Common-law/In a relationship	60 (54.6%)	62 (40.0%)	0.019
	Single/Separated/Divorced/Widow/Other relationship status	50 (45.5%)	93 (60.0%)	
Sexual orientation				
	Heterosexual	103 (93.6%)	134 (86.5%)	0.061
	LGBTQ	7 (6.4%)	21 (13.6%)	
HCV co-infection				
	Yes	24 (21.8%)	60 (38.7%)	0.004
	No	86 (78.2%)	95 (61.3%)	
Diagnosis of mental health condition by care provider				
	Yes	36 (32.7%)	76 (49.0%)	0.01
	No	71 (64.5%)	77 (49.7%)	
	Other+	*	*	
History of injection drug use				
	Yes	26 (23.6%)	60 (38.7%)	0.009
	No	84 (76.4%)	94 (60.7%)	
	Other+	0 (0)	*	
Era of cART initiation				
	Before Year 2000	17 (15.5%)	45 (29.0%)	0.014
	Year 2000 or after	81 (73.6%)	98 (63.2%)	
	Other+	12 (10.9%)	12 (7.7%)	
Years living with HIV, at interview				
	Less than 6 years	16 (14.5%)	16 (10.3%)	0.277
	6 to 14 years	57 (51.8%)	73 (47.1%)	
	More than 14 years	37 (33.6%)	66 (42.6%)	
Currently pregnant, at interview				
	Yes	8 (7.27%)	7 (4.5%)	0.342
	No	101 (91.8%)	146 (94.2%)	
	Other +	*	*	
Feeling when found out about current/recent pregnancy				
	Very happy/happy to be pregnant	100 (90.9%)	67 (43.2%)	<0.001
	Not sure/Unhappy/Very unhappy to be pregnant	8 (7.3%)	87 (56.1%)	
	Other +	*	*	
Time during the current/recent pregnancy				
	One of the happiest times of my life/A happy time with a few problems	91 (82.7%)	57 (36.8%)	<0.001
	A moderately hard/Very hard/Worst time	18 (16.4%)	93 (60.0%)	
	Other +	*	5 (3.2%)	
HIV status of the other biological parent				
	HIV positive	36 (32.7%)	38 (24.5%)	0.016
	HIV negative	63 (57.3%)	80 (51.6%)	
	Unknown HIV status	10 (9.1%)	34 (21.9%)	
	Other +	*	*	
Intend to become pregnant in the future				
	Yes	31 (28.2%)	30 (19.6%)	0.143
	No	51 (46.4%)	78 (50.3%)	
	Other+	28 (25.5%)	47 (30.3%)	
On cART before pregnancy occurred				
	Yes	88 (80.0%)	117 (75.5%)	0.359
	No	21 (19.1%)	37 (23.9%)	
	Other+	*	*	
Pregnancy outcome				
	Single/multiple live birth	83 (75.5%)	86 (55.5%)	<0.001
	Miscarriage/Stillbirth/Ectopic pregnancy	17 (15.5%)	18 (11.6%)	
	Pregnancy termination	*	44 (28.4%)	
	Current pregnancy at interview^	8 (7.3%)	7 (4.5%)	
HIV test result of baby				
	HIV positive	0 (0)	*	0.999
	HIV negative	80 (72.7%)	81 (52.3%)	
	Testing underway	*	*	
	Other/No live birth	29 (26.4%)	71 (45.8%)	
		Median (IQR)	Median	P-value
Age at interview date		38 (34–43)	37 (33–43)	0.674
Duration of HIV Diagnosis (in years)		12.0 (7.8–16.1)	13.2 (8.2–18.6)	0.146
Number of previous pregnancies		3 (2–4)	3 (2–5)	0.967
Age at conception (n = 255)		32 (29–36)	31 (27–35)	0.027
Number of years to first pregnancy after HIV diagnosis		3.6 (1.5–6.1)	3.1 (1.2–5.1)	0.331

Notes: LGBTTQ, lesbian, gay, bisexual, trans, two-spirit or queer; cART, combination antiretroviral therapy

*Cells indicated where n<5 and excluded from p-value analysis

^^^ Excluded from p-value analysis

^+^Inclusive of don’t know, prefer not to answer, and other responses

With respect to pregnancy experiences, women with an unintended vs. intended recent/current pregnancy were more likely to report feeling unhappy about the referent pregnancy (56.1% vs. 7.3%) and identifying the pregnancy experience as ‘one of the worst times of [her] life’ (60.0% vs. 16.4%). Women who reported a recent/current pregnancy as unintended vs. intended were also more likely to report pregnancy termination (28.4% vs. 1.8%).

In multivariable analyses (Table 4), factors independently associated with a most recent/current unintended pregnancy included being currently single (AOR: 1.94, 95% CI: 1.10, 3.42), being younger at time of pregnancy (AOR: 0.95 per year increase, 95% CI: 0.90, 0.99), and being born in Canada (vs outside of Canada) (AOR: 2.76, 95% CI: 1.55, 4.92).

**Table 4:** Univariate and Multivariable model of factors associated with unintended (vs intended) pregnancy for most recent or current pregnancy, n = 265.

Variable	Response	Unadjusted Odds Ratio (95% CI)	Adjusted Odds Ratio (aOR) (95% CI)
Ethnicity			
	White	Reference	Not selected
	Indigenous	1.14 (0.53, 2.47)	
	African/Caribbean/Black	0.40 (0.23, 0.71)	
	Other ethnicities	0.54 (0.19, 1.52)	
Born in Canada			
	No	Reference	Reference
	Yes	2.75 (1.66, 4.56)	2.76 (1.55, 4.92)
Current legal relationship status			
	Married/Common-law	Reference	Reference
	Single/Divorced/Widow	1.80 (1.098, 2.950)	1.94 (1.10, 3.42)
Diagnosis of mental health condition by care provider			
	No	Reference	Reference
	Yes	1.95 (1.17, 3.25)	1.53 (0.85, 2.75)
Age at conception, per one year increase (n = 255)		0.95 (0.90, 0.99)	0.95 (0.90, 0.99)
HIV status of the other parent			
	HIV-positive	Reference	Not selected
	HIV-negative/unknown	1.48 (0.86, 2.55)	
cART era			
	≥2000	Reference	Not selected
	<2000	2.19 (1.16, 4.11)	

## Discussion

Among women living with HIV in Canada, we found that approximately one in four women reported pregnancy after HIV diagnosis and that pregnancy was more frequent in the modern cART era and with uptake of cART. Pregnancy incidence rates observed in this study are lower than rates observed in other studies of WLWH in other global settings [11, 15, 49, 50], however is consistent with a lower pregnancy rate in the general Canadian population (54.6 per 1000 women) [51].

Approximately 60% of pregnancies after HIV diagnosis among women in this study were reported as unintended and the prevalence varied by key socio-demographic characteristics. The high observed prevalence of unintended pregnancy is consistent with estimates from a cohort of WLWH in Ontario, where 57% of women reported that their most recent pregnancy was unplanned [12] and comparable to the high rates of unintended pregnancies observed among WLWH in other high-resource settings [52, 53]. Research conducted in Sub-Saharan Africa, including Botswana and South Africa demonstrate that 37% to 69% of pregnancies among WLWH are unintended [20, 54–56]. In the United States, approximately 69% of a total of 620 pregnancy events among a sample of WLWH from 26 different regions were determined to be unplanned [53].

This study is the first of its kind to look at the impact of cART uptake and pregnancy rates among WLWH in Canada. After decades of declining numbers of women becoming pregnant after their HIV diagnosis [29, 36], we observed that WLWH on cART are more likely to become pregnant compared to those not on cART prior to pregnancy, with incidence rates nearly three times of that observed among WLWH not on cART. It must be noted that the majority of pregnancy events in our sample (86.5%) occurred after the year 2000, and these WLWH were more likely to become pregnant, and sooner after HIV diagnosis, compared to women in the earlier treatment era. It is important to note that while a previous Canadian study found no association between use of cART and fertility intentions, our study shows an association between cART use and pregnancy events, further highlighting the importance of addressing unintended pregnancy rates for all WLWH [17].

Previous studies have shown increases in pregnancy incidence after cART initiation [15, 20, 23, 57]. Observed increases in pregnancies for WLWH on cART are likely due to a combination of behavioural and biological factors including, increased life expectancy, increase in sexual activity, and improved health and fertility status [31, 58, 59]. While some research has stated that uptake of cART may improve engagement in care and thus increase uptake of contraceptives [60], our data suggests that a gap in family planning specific to WLWH exists given that 60% of pregnancies are considered unintended. Our data indicate that pregnancy is more common with cART uptake and current trends suggest future increases, although the role of contraceptive uptake will need to be more thoroughly investigated. While we did not capture lifetime contraceptive use patterns, data on contraceptive use at baseline among sexually active women enrolled in CHIWOS reveal that 27.4% had not used an effective contraceptive method in the previous six months [61]. Among those women who did report using contraception, nearly half relied on the male condom [61]. This emerging work, as well as our finding around the high prevalence of unintended pregnancy, underscores the need for improved integration of family planning care as part of comprehensive HIV care for women [11, 62, 63].

Our study suggests that more than half of pregnancies among WLWH are unintended similar to rates observed in other studies of WLWH, which range from 51–90% [32, 34]. Such rates are significantly higher than recent estimates among Canadian women of reproductive age in the general population, among whom an estimated 27% of pregnancies are unintended [64]. Pregnancy termination occurred in one in five pregnancies among WLWH in our sample. Consistent with earlier findings, women in our sample were significantly more likely to terminate an unintended pregnancies compared to a reported intended pregnancy [20, 31, 65].

There are several limitations to this study. Our analyses rely on data collected via questionnaire suggesting recall bias is likely present. The retrospective data collection on lifetime history of pregnancy did not allow for an assessment of important behaviours prior to or during pregnancy including contraceptive use at the time of each reported pregnancy, relationship status, smoking, substance use and other socio-behavioural factors. Moreover, with a sensitive outcome of interest being collected retrospectively, there may be some misclassification bias due to shifting perceptions of pregnancy intention in relation to the subsequent outcome of that pregnancy. However, research has suggested that retrospectively collecting data on pregnancy intention likely does not lead to misleading estimates of the prevalence of unintended pregnancies [66], however this has not been validated in a context with WLWH where there may still be significant stigma attached to becoming pregnant or intending to become pregnant. In an attempt to mitigate the influence of bias, questionnaires were administered by trained peer research associates, who were also living with HIV, and were able to guide participants through their pregnancy history. The authors believe this may better support accurate data collection (relative to self-administered or non-peer administered surveys). As well, participants were aware that they were being interviewed by women who shared a lived experience of living with HIV, which likely reduced social desirability bias.

Our rates of pregnancy may be underestimating fertility rates of WLWH as our cohort is older and may not represent all women who are currently living with HIV of reproductive age. Moreover, post-menopausal women who were under 45 years of age were not appropriately censored in this analysis since we had no date of spontaneous menopause As such, our at-risk time may be inflated and our estimates of pregnancy incidence may be lower than actual rates. Although we excluded 127 women who were 45 years old or older at the time of HIV diagnosis, censoring women at 45 was deliberately done to most accurately capture those likely to become pregnant. We found only fewer than 5 women in our sample over the age of 45 who had pregnancy events after HIV diagnosis. In addition, we did not have detailed pregnancy data after eight lifetime pregnancy events and 28 (2.4%) of WLWH in our sample reported more than eight pregnancies. Despite these limitations, our study provides the most comprehensive picture of pregnancy incidence among WLWH in Canada with over 1400 participants and on-going data collection that providing longitudinal data on sexual and reproductive health outcomes among WLWH.

Given known risks of unintended pregnancy to maternal, partner, and perinatal health, our findings underscore the need for comprehensive women-centered sexual and reproductive health programming for WLWH, including provision of effective contraceptive options and pregnancy planning support, and access to safe termination services. In the modern cART era, WLWH must have access to comprehensive reproductive health care and support that extends beyond concerns of sexual and perinatal HIV transmission. These changing reproductive health patterns, including more WLWH becoming pregnant and a high prevalence of unintended pregnancy, have significant implications for women’s overall well-being. Promotion of reproductive health care, supporting women’s reproductive autonomy, decisions and desires, and enhancing access to effective contraceptive options alongside safe, non-coercive pregnancy termination services should be available in conjunction with evolving HIV care and improved therapeutic options for WLWH.
